# Association between chronic rhinosinusitis and pneumonia: a longitudinal follow-up study using a national health screening cohort

**DOI:** 10.1038/s41598-022-09552-8

**Published:** 2022-03-31

**Authors:** Jee Hye Wee, Chanyang Min, Hahn Jin Jung, Min Woo Park, Bumjung Park, Hyo Geun Choi

**Affiliations:** 1 Department of Otorhinolaryngology-Head & Neck Surgery, Hallym University College of Medicine, Hallym University Sacred Heart Hospital, 22, Gwanpyeong-ro 170-beon-gil, Dongan-gu, Anyang, Gyeonggi-do 14068 Korea; 2 Hallym Data Science Laboratory, Hallym University College of Medicine, Anyang, Korea; 3 Graduate School of Public Health, Seoul National University, Seoul, Korea; 4 Department of Otorhinolaryngology-Head & Neck Surgery, Chungbuk National University College of Medicine, Chungbuk National University Hospital, Cheongju, Korea; 5 Department of Otorhinolaryngology-Head & Neck Surgery, Kangdong Sacred Heart Hospital, Seoul, Korea

**Keywords:** Inflammation, Respiratory tract diseases, Risk factors

## Abstract

This study was aimed to compare the risk of pneumonia between patients with chronic rhinosinusitis (CRS) and those without CRS (control) in a Korean population. The population aged 40 years or over was included from the Korean National Health Insurance Service-Health Screening Cohort. Participants with CRS (n = 6393) and controls (n = 25,572) were selected by 1:4 matching for age, sex, income, region of residence, and history of pneumonia for the previous 1 year. The index date (ID) of the controls was set as the treatment date of their matched CRS participants. The incidence of pneumonia after the ID was measured from 2003 to 2015. Simple and multiple linear regressions were performed to calculate estimated values (EVs) and 95% confidence intervals (CIs) for 1-y post-ID pneumonia, 2-y post-ID pneumonia, and 3-y post-ID pneumonia in CRS participants compared to controls. Statistical significance was noted in the 3-y post-ID period (EV = 0.017, 95% CI = 0.002–0.031, *P* = 0.030). In the subgroup analyses according to age and sex, statistical significance was seen in the younger age group (< 60 years old) in the 3-y post-ID period and in the female group in the 1-y and 3-y post-ID periods. This study revealed an increased risk for pneumonia following a diagnosis of CRS.

## Introduction

Pneumonia is a form of acute respiratory infection caused by viruses, bacteria, and fungi and a major cause of morbidity and mortality globally. According to the World Health Organization (WHO)’s 2019 Global Health Estimates, pneumonia and other lower respiratory infections were ranked as the fourth leading cause of death and disability-adjusted life years^1^. In Korea, deaths due to pneumonia have continued to increase since 2000, and in 2019, the death rate for pneumonia stood at 45.1 per 100,000 population, ranking as the third leading cause of deaths^2^. In a recent study in Korea, the hospitalization rate due to bacterial pneumonia was reported to be 161.5 per 10,000 population over the age of 65^3^. Several risk factors, such as diabetes, cardiovascular disease, smoking, asthma, and chronic obstructive pulmonary disease (COPD), have been shown to increase the likelihood of the development of pneumonia^4^.

Chronic rhinosinusitis (CRS) is a chronic upper airway inflammation that can greatly influence the health-related quality of life of patients and the socioeconomic burden^5^. Several epidemiological studies have shown that CRS is associated with lower airway diseases such as asthma^6^, bronchiectasis^7^, and COPD^8^. This interdependence of the upper and lower respiratory tracts has led to the concept of the “unified airway”, which considers the upper and lower airways to be a single functional unit^9^.

Despite the correlations between the upper and lower airways, few studies have evaluated the association of CRS and pneumonia. A retrospective study using X-ray findings reported that the frequency of sinusitis was found to be 84% among patients with pneumonia^10^. A previous study in the U.S. showed an association between community-acquired pneumonia and CRS with sphenoid involvement (odds ratio [OR] = 19.76, 95% confidence interval [CI] = 8.78–44.47)^11^. Furthermore, population-based studies evaluating the risk of pneumonia in patients with CRS are lacking.

This study aimed to compare the incidence of pneumonia between patients with CRS and those without CRS (control) using a national sample cohort from the Korean population. An understanding of the association between CRS and pneumonia will contribute to disease prevention.

## Results

Table 1 shows the general characteristics of the participants. Age, sex, income, region of residence, and 1-y pre-index date (ID) pneumonia showed no difference between the CRS and control groups due to matching (standardized difference = 0.00). Total cholesterol level, systolic blood pressure (SBP)/diastolic blood pressure (DBP), fasting glucose level, obesity, smoking status, alcohol consumption, and Charlson Comorbidity Index (CCI) scores were similar in the CRS total group and controls (standardized difference < 0.20), while the prevalence rates of asthma and COPD were different between the two groups (standardized difference ≥ 0.20).

**Table 1:** General characteristics of participants.

Characteristics	Total participants		
	CRS total (n = 6,393)	Control (n = 25,572)	Standardized difference
**CRS with nasal polyps & control (n, %)**	3304 (51.7)	13,216 (51.7)	
**CRS without nasal polyps & control (n, %)**	3089 (48.3)	12,356 (48.3)	
**Age (years old, n, %)**			0.00
40–44	424 (6.6)	1696 (6.6)	
45–49	1224 (19.2)	4896 (19.2)	
50–54	1419 (22.2)	5676 (22.2)	
55–59	1259 (19.7)	5036 (19.7)	
60–64	933 (14.6)	3732 (14.6)	
65–69	626 (9.8)	2504 (9.8)	
70–74	343 (5.4)	1372 (5.4)	
75–79	126 (2.0)	504 (2.0)	
80–84	31 (0.5)	124 (0.5)	
≥ 85	8 (0.1)	32 (0.1)	
**Sex (n, %)**			0.00
Male	3880 (60.7)	15,520 (60.7)	
Female	2513 (39.3)	10,052 (39.3)	
**Income (n, %)**			0.00
1 (lowest)	753 (11.8)	3012 (11.8)	
2	767 (12.0)	3068 (12.0)	
3	981 (15.3)	3924 (15.3)	
4	1355 (21.2)	5420 (21.2)	
5 (highest)	2537 (39.7)	10,148 (39.7)	
**Region of residence (n, %)**			0.00
Urban	2999 (46.9)	11,996 (46.9)	
Rural	3394 (53.1)	13,576 (53.1)	
**Total cholesterol level (mg/dL, mean, SD)**	198.0 (37.0)	199.4 (38.0)	0.04
**SBP (mmHg, mean, SD)**	125.5 (16.4)	126.8 (17.1)	0.07
**DBP (mmHg, mean, SD)**	78.7 (10.8)	79.2 (11.1)	0.05
**Fasting blood glucose level (mg/dL, mean, SD)**	98.5 (31.4)	100.1 (33.1)	0.05
**Obesity**^**a**^** (n, %)**			0.07
Underweight	111 (1.7)	549 (2.2)	
Normal	2061 (32.2)	8755 (34.2)	
Overweight	1906 (29.8)	7124 (27.9)	
Obese I	2144 (33.5)	8349 (32.7)	
Obese II	171 (2.7)	795 (3.1)	
**Smoking status (n, %)**			0.10
Nonsmoker	4285 (67.0)	16,685 (65.3)	
Past smoker	841 (13.2)	2829 (11.1)	
Current smoker	1267 (19.8)	6058 (23.7)	
**Alcohol consumption (n, %)**			0.03
< 1 time a week	4424 (69.2)	17,287 (67.6)	
≥ 1 time a week	1969 (30.8)	8,285 (32.4)	
**CCI score (score, n, %)**			0.10
0	4412 (69.0)	18,686 (73.1)	
1	905 (14.2)	3012 (11.8)	
2	527 (8.2)	1754 (6.9)	
≥ 3	549 (8.6)	2120 (8.3)	
**Asthma (n, %)**	1910 (29.9)	4175 (16.3)	0.33
**COPD (n, %)**	756 (11.8)	1593 (6.2)	0.20
**1-y pre-ID pneumonia (n, %)**			0.00
0 times	6209 (97.1)	24,836 (97.1)	
≥ 1 time	184 (2.9)	736 (2.9)	
**Post-ID pneumonia (mean, SD)**			
First year-periods	0.08 (0.67)	0.05 (0.54)	0.05
Second year-periods	0.08 (0.57)	0.05 (0.58)	0.05
Third year-periods	0.08 (0.65)	0.05 (0.52)	0.06

CCI, Charlson comorbidity index; COPD, chronic obstructive pulmonary disease; CRS, chronic rhinosinusitis; DBP, diastolic blood pressure; 1-y pre-ID pneumonia, pneumonia history from the date of first diagnosis of total CRS (index date) to the date before 1-year periods; Post-ID pneumonia, the number of pneumonia diagnosis from the index date to the date after certain periods; SBP, systolic blood pressure; SD, standard deviation.

^a^Obesity (BMI, body mass index, kg/m^2^) was categorized as < 18.5 (underweight), ≥ 18.5 to < 23 (normal), ≥ 23 to < 25 (overweight), ≥ 25 to < 30 (obese I), and ≥ 30 (obese II).

The adjusted estimated value (EV) of the incidence of post-ID pneumonia did not reach statistical significance at 1-y (P = 0.059) and 2-y (*P* = 0.310) post-ID. However, the adjusted EV showed statistical significance at 3-y post-ID (EV = 0.017, 95% CI = 0.002–0.031, P = 0.030) (Table 2).

**Table 2:** Simple and multiple linear regression model (estimated value [95% confidence intervals]) for post index date of pneumonia (post-ID pneumonia) periods in total chronic rhinosinusitis compared to control group and subgroup analysis according to age and sex.

Characteristics	Mean ± SD in CRS total group	Mean ± SD in control group	Linear regression of CRS for pneumonia					
			Simple^b^	*P* value	Model 1^b,^^c^	*P* value	Model 2^b,^^d^	*P* value
**Total participants (n = 31,965)**								
1-y post-ID pneumonia	0.08 ± 0.67	0.05 ± 0.54	0.030 (0.015 to 0.046)	< 0.001^a^	0.008 (− 0.007 to 0.024)	0.305	0.015 (− 0.001 to 0.030)	0.059
2-y post-ID pneumonia	0.08 ± 0.57	0.05 ± 0.58	0.027 (0.011 to 0.042)	0.001^a^	0.004 (− 0.012 to 0.019)	0.651	0.008 (− 0.008 to 0.024)	0.310
3-y post-ID pneumonia	0.08 ± 0.65	0.05 ± 0.52	0.033 (0.018 to 0.048)	< 0.001^a^	0.013 (− 0.002 to 0.028)	0.079	0.017 (0.002 to 0.031)	0.030^a^
**Age < 60 years old (n = 21,630)**								
1-y post-ID pneumonia	0.05 ± 0.51	0.03 ± 0.36	0.023 (0.010 to 0.036)	0.001^a^	0.009 (− 0.005 to 0.022)	0.204	0.012 (− 0.001 to 0.025)	0.065
2-y post-ID pneumonia	0.05 ± 0.44	0.03 ± 0.35	0.021 (0.009 to 0.034)	0.001^a^	0.009 (− 0.003 to 0.022)	0.141	0.012 (− 0.001 to 0.024)	0.060
3-y post-ID pneumonia	0.06 ± 0.61	0.03 ± 0.33	0.036 (0.022 to 0.049)	< 0.001^a^	0.023 (0.009 to 0.036)	0.001^a^	0.025 (0.011 to 0.038)	< 0.001^a^
**Age ≥ 60 years old (n = 10,335)**								
1-y post-ID pneumonia	0.15 ± 0.91	0.10 ± 0.80	0.045 (0.006 to 0.085)	0.024^a^	0.014 (− 0.026 to 0.053)	0.498	0.025 (− 0.014 to 0.063)	0.206
2-y post-ID pneumonia	0.14 ± 0.79	0.10 ± 0.88	0.038 (− 0.003 to 0.079)	0.071	0.000 (− 0.041 to 0.041)	0.997	0.008 (− 0.033 to 0.048)	0.717
3-y post-ID pneumonia	0.12 ± 0.73	0.09 ± 0.77	0.027 (− 0.009 to 0.064)	0.144	0.000 (− 0.037 to 0.037)	0.999	0.005 (− 0.032 to 0.041)	0.798
**Males (n = 19,400)**								
1-y post-ID pneumonia	0.07 ± 0.57	0.06 ± 0.56	0.018 (− 0.001 to 0.038)	0.066	− 0.005 (− 0.024 to 0.015)	0.638	0.001 (− 0.018 to 0.020)	0.943
2-y post-ID pneumonia	0.08 ± 0.58	0.05 ± 0.59	0.024 (0.004 to 0.044)	0.021^a^	0.000 (− 0.020 to 0.021)	0.988	0.004 (− 0.016 to 0.024)	0.711
3-y post-ID pneumonia	0.08 ± 0.61	0.05 ± 0.57	0.030 (0.010 to 0.050)	0.003^a^	0.011 (− 0.010 to 0.031)	0.303	0.013 (− 0.007 to 0.033)	0.202
**Females (n = 12,565)**								
1-y post-ID pneumonia	0.10 ± 0.80	0.05 ± 0.52	0.048 (0.023 to 0.074)	< 0.001^a^	0.028 (0.003 to 0.054)	0.030^a^	0.037 (0.012 to 0.062)	0.004^a^
2-y post-ID pneumonia	0.08 ± 0.57	0.04 ± 0.56	0.031 (0.006 to 0.056)	0.013^a^	0.008 (− 0.017 to 0.033)	0.535	0.014 (− 0.011 to 0.038)	0.278
3-y post-ID pneumonia	0.08 ± 0.71	0.04 ± 0.43	0.038 (0.016 to 0.060)	0.001^a^	0.018 (− 0.004 to 0.041)	0.103	0.022 (0.000 to 0.044)	0.046^a^

Abbreviations: CCI, Charlson comorbidity index; COPD, chronic obstructive pulmonary disease; DBP, diastolic blood pressure; CRS, chronic rhinosinusitis; 1-y pre-ID pneumonia, pneumonia history from the date of CRS treatment (index date) to the date before 1-year periods; Post-ID pneumonia, the number of pneumonia diagnosis from the index date to the date after certain periods; SBP, systolic blood pressure; SD, standard deviation.

^a^Linear regression model, Significance at *P* < 0.05.

^b^Models were stratified by age, sex, income, and region of residence.

^c^A model 1 was adjusted for obesity, smoking, alcohol consumption, total cholesterol, SBP, DBP, fasting blood glucose, CCI scores, asthma, and COPD.

^d^A model 2 was adjusted for the model 1 plus 1-y pre-ID pneumonia.

In the subgroup analyses according to age and sex, statistical significance was seen in the younger age group (< 60 years old) at 3-y post-ID (EV = 0.025, 95% CI = 0.011–0.038, *P* < 0.001) and the female group at the 1-y (EV = 0.037, 95% CI = 0.012–0.062, *P* = 0.004) and 3-y (EV = 0.022, 95% CI = 0.000–0.044, *P* = 0.046) post-ID periods (Table 2).

When analyzing the CRS with nasal polyps (CRSwNP) (Table 3) and CRS without nasal polyps (CRSsNP) (Table 4) groups separately, an association between CRS and pneumonia was found in the 1-y (EV = 0.035, 95% CI = 0.010–0.060, *P* = 0.007) and 3-y (EV = 0.037, 95% CI = 0.014–0.061, *P* = 0.002) post-ID periods in the CRSsNP group only.

**Table 3:** Simple and multiple linear regression model (estimated value [95% confidence intervals]) for post index date of pneumonia (post-ID pneumonia) periods in chronic rhinosinusitis with nasal polyp compared to control group and subgroup analysis according to age and sex.

Characteristics	Mean ± SD in CRS with nasal polyp group	Mean ± SD in control group	Linear regression of CRS with nasal polyp for pneumonia					
			Simple^b^	*P* value	Model 1^b,^^c^	*P* value	Model 2^b,d^	*P* value
**Total participants (n = 16,520)**								
1-y post-ID pneumonia	0.06 ± 0.44	0.05 ± 0.48	0.011 (− 0.007 to 0.028)	0.240	− 0.008 (− 0.025 to 0.010)	0.398	− 0.004 (− 0.021 to 0.013)	0.653
2-y post-ID pneumonia	0.06 ± 0.45	0.05 ± 0.57	0.013 (− 0.008 to 0.033)	0.232	− 0.004 (− 0.025 to 0.017)	0.710	− 0.001 (− 0.022 to 0.020)	0.920
3-y post-ID pneumonia	0.05 ± 0.47	0.04 ± 0.49	0.009 (− 0.009 to 0.028)	0.336	− 0.004 (− 0.023 to 0.014)	0.643	− 0.003 (− 0.021 to 0.016)	0.786
**Age < 60 years old (n = 11,705)**								
1-y post-ID pneumonia	0.04 ± 0.36	0.03 ± 0.33	0.010 (− 0.005 to 0.026)	0.176	− 0.002 (− 0.017 to 0.014)	0.822	0.002 (− 0.013 to 0.017)	0.811
2-y post-ID pneumonia	0.03 ± 0.30	0.02 ± 0.32	0.009 (− 0.006 to 0.023)	0.234	− 0.003 (− 0.017 to 0.011)	0.674	− 0.001 (− 0.015 to 0.013)	0.910
3-y post-ID pneumonia	0.04 ± 0.43	0.02 ± 0.27	0.021 (0.007 to 0.035)	0.004^a^	0.012 (− 0.002 to 0.026)	0.104	0.013 (− 0.001 to 0.028)	0.060
**Age ≥ 60 years old (n = 4815)**								
1-y post-ID pneumonia	0.10 ± 0.59	0.09 ± 0.72	0.011 (− 0.037 to 0.058)	0.663	− 0.014 (− 0.062 to 0.034)	0.566	− 0.010 (− 0.057 to 0.037)	0.675
2-y post-ID pneumonia	0.12 ± 0.68	0.10 ± 0.92	0.022 (− 0.040 to 0.084)	0.484	0.002 (− 0.060 to 0.064)	0.939	0.006 (− 0.055 to 0.068)	0.843
3-y post-ID pneumonia	0.07 ± 0.55	0.09 ± 0.81	− 0.019 (− 0.073 to 0.035)	0.490	− 0.036 (− 0.090 to 0.018)	0.192	− 0.034 (− 0.088 to 0.020)	0.217
**Males (n = 10,880)**								
1-y post-ID pneumonia	0.05 ± 0.42	0.05 ± 0.52	0.005 (− 0.018 to 0.028)	0.659	− 0.013 (− 0.036 to 0.010)	0.282	− 0.010 (− 0.033 to 0.013)	0.391
2-y post-ID pneumonia	0.06 ± 0.47	0.05 ± 0.58	0.015 (− 0.011 to 0.041)	0.254	− 0.002 (− 0.028 to 0.024)	0.884	0.000 (− 0.026 to 0.026)	0.998
3-y post-ID pneumonia	0.05 ± 0.46	0.05 ± 0.55	0.002 (− 0.023 to 0.027)	0.869	− 0.012 (− 0.037 to 0.013)	0.348	− 0.010 (− 0.035 to 0.014)	0.413
**Females (n = 5640)**								
1-y post-ID pneumonia	0.06 ± 0.48	0.04 ± 0.38	0.021 (− 0.005 to 0.047)	0.118	0.003 (− 0.023 to 0.030)	0.798	0.009 (− 0.017 to 0.035)	0.493
2-y post-ID pneumonia	0.05 ± 0.39	0.04 ± 0.55	0.008 (− 0.026 to 0.042)	0.654	− 0.010 (− 0.044 to 0.024)	0.566	− 0.005 (− 0.039 to 0.029)	0.772
3-y post-ID pneumonia	0.06 ± 0.50	0.04 ± 0.37	0.023 (− 0.003 to 0.049)	0.088	0.010 (− 0.016 to 0.036)	0.449	0.012 (− 0.014 to 0.039)	0.355

Abbreviations: CCI, Charlson comorbidity index; COPD, chronic obstructive pulmonary disease; DBP, diastolic blood pressure; CRS, chronic rhinosinusitis; 1-y pre-ID pneumonia, pneumonia history from the date of CRS treatment (index date) to the date before 1-year periods; Post-ID pneumonia, the number of pneumonia diagnosis from the index date to the date after certain periods; SBP, systolic blood pressure; SD, standard deviation.

^a^Linear regression model, Significance at *P* < 0.05.

^b^Models were stratified by age, sex, income, and region of residence.

^c^A model 1 was adjusted for obesity, smoking, alcohol consumption, total cholesterol, SBP, DBP, fasting blood glucose, CCI scores, asthma, and COPD.

^d^A model 2 was adjusted for the model 1 plus 1-y pre-ID pneumonia.

**Table 4:** Simple and multiple linear regression model (estimated value [95% confidence intervals]) for post index date of pneumonia (post-ID pneumonia) periods in chronic rhinosinusitis without nasal polyp compared to control group and subgroup analysis according to age and sex.

Characteristics	Mean ± SD in CRS without nasal polyp group	Mean ± SD in control group	Linear regression of CRS without nasal polyp for pneumonia					
			Simple^b^	*P* value	Model 1^bc^	*P* value	Model 2^bd^	*P* value
**Total participants (n = 15,445)**								
1-y post-ID pneumonia	0.11 ± 0.85	0.06 ± 0.60	0.051 (0.025 to 0.077)	< 0.001^a^	0.025 (− 0.001 to 0.051)	0.056	0.035 (0.010 to 0.060)	0.007^a^
2-y post-ID pneumonia	0.09 ± 0.68	0.05 ± 0.59	0.042 (0.018 to 0.066)	0.001^a^	0.011 (− 0.013 to 0.035)	0.367	0.017 (− 0.006 to 0.041)	0.155
3-y post-ID pneumonia	0.11 ± 0.80	0.05 ± 0.54	0.059 (0.035 to 0.082)	< 0.001^a^	0.033 (0.009 to 0.057)	0.007^a^	0.037 (0.014 to 0.061)	0.002^a^
**Age < 60 years old (n = 9925)**								
1-y post-ID pneumonia	0.07 ± 0.65	0.03 ± 0.39	0.038 (0.015 to 0.060)	0.001^a^	0.021 (− 0.002 to 0.044)	0.068	0.025 (0.003 to 0.047)	0.027^a^
2-y post-ID pneumonia	0.06 ± 0.56	0.03 ± 0.38	0.036 (0.016 to 0.057)	0.001^a^	0.023 (0.002 to 0.044)	0.030^a^	0.026 (0.005 to 0.047)	0.014^a^
3-y post-ID pneumonia	0.09 ± 0.77	0.03 ± 0.39	0.054 (0.030 to 0.078)	< 0.001^a^	0.035 (0.011 to 0.060)	0.005^a^	0.038 (0.013 to 0.062)	0.003^a^
**Age ≥ 60 years old (n = 5520)**								
1-y post-ID pneumonia	0.19 ± 1.12	0.11 ± 0.86	0.075 (0.015 to 0.136)	0.014^a^	0.039 (− 0.022 to 0.099)	0.212	0.057 (− 0.001 to 0.115)	0.056
2-y post-ID pneumonia	0.15 ± 0.87	0.10 ± 0.84	0.052 (− 0.003 to 0.108)	0.066	− 0.003 (− 0.058 to 0.052)	0.917	0.008 (− 0.046 to 0.063)	0.771
3-y post-ID pneumonia	0.16 ± 0.85	0.09 ± 0.74	0.068 (0.018 to 0.118)	0.008^a^	0.035 (− 0.015 to 0.085)	0.174	0.042 (− 0.008 to 0.092)	0.097
**Males (n = 8,520)**								
1-y post-ID pneumonia	0.10 ± 0.72	0.06 ± 0.60	0.035 (0.002 to 0.068)	0.037^a^	0.006 (− 0.027 to 0.039)	0.730	0.015 (− 0.017 to 0.047)	0.367
2-y post-ID pneumonia	0.09 ± 0.68	0.06 ± 0.60	0.036 (0.003 to 0.068)	0.032^a^	0.001 (− 0.032 to 0.033)	0.958	0.007 (− 0.025 to 0.039)	0.677
3-y post-ID pneumonia	0.12 ± 0.76	0.06 ± 0.59	0.066 (0.033 to 0.099)	< 0.001^a^	0.040 (0.007 to 0.073)	0.019^a^	0.044 (0.011 to 0.077)	0.010^a^
**Females (n = 6,925)**								
1-y post-ID pneumonia	0.13 ± 0.98	0.06 ± 0.60	0.071 (0.030 to 0.112)	0.001^a^	0.050 (0.008 to 0.091)	0.019^a^	0.060 (0.020 to 0.100)	0.004^a^
2-y post-ID pneumonia	0.10 ± 0.69	0.05 ± 0.57	0.050 (0.015 to 0.085)	0.005^a^	0.023 (− 0.013 to 0.058)	0.211	0.029 (− 0.006 to 0.064)	0.104
3-y post-ID pneumonia	0.10 ± 0.85	0.05 ± 0.48	0.050 (0.016 to 0.084)	0.004^a^	0.024 (− 0.010 to 0.058)	0.163	0.029 (− 0.004 to 0.063)	0.088

CCI, Charlson comorbidity index; COPD, chronic obstructive pulmonary disease; DBP, diastolic blood pressure; CRS, chronic rhinosinusitis; 1-y pre-ID pneumonia, pneumonia history from the date of CRS treatment (index date) to the date before 1-year periods; Post-ID pneumonia, the number of pneumonia diagnosis from the index date to the date after certain periods; SBP, systolic blood pressure; SD, standard deviation.

^a^Linear regression model, Significance at *P* < 0.05.

^b^Models were stratified by age, sex, income, and region of residence.

^c^A model 1 was adjusted for obesity, smoking, alcohol consumption, total cholesterol, SBP, DBP, fasting blood glucose, CCI scores, asthma, and COPD.

^d^A model 2 was adjusted for the model 1 plus 1-y pre-ID pneumonia.

## Discussion

This study showed the association between CRS and pneumonia. This association was seen only in the younger age, female sex, and CRSsNP subgroups.

The concept of “united airway disease” or “one linked airway disease” has emerged, and allergic rhinitis, sinusitis, and asthma are expressed in different parts of the respiratory tract as one pathological process that is inflammatory in nature^12^. In addition, the concept has expanded beyond asthma, and an association with CRS has been reported in other lung diseases, including bronchiectasis^7,13^ and COPD^8^. In a systematic review, the pooled prevalence of CRS in adults with bronchiectasis was 62%, and CRS was associated with a greater degree of bronchiectasis severity, poorer quality of life, reduction in olfactory detection, and elevated levels of inflammatory markers^7^. A large retrospective study in a U.S. population found that almost half of the patients with bronchiectasis had comorbid CRS^13^. In a Taiwanese population-based study, COPD was associated with CRSsNP^8^.

On the other hand, there have been few studies on the association between CRS and pneumonia. Previous studies have mainly reported the incidence of sinusitis and pneumonia in intensive care unit (ICU) patients^14–16^. A meta-analysis on ventilator-associated sinusitis showed that 41% of patients with ICU-derived sinusitis also developed hospital-acquired pneumonia^17^. They showed that the same pathogen was isolated from culture specimens of the lung and sinuses in 59% of patients^17^. However, few studies have evaluated the association of CRS and pneumonia in the general population.

We hypothesize that there are several possible mechanisms underlying the association between CRS and pneumonia. First, the nasal cavities constitute the first barrier of the whole airway system that has immunological functions against bacteria, viruses, and other pathogens. Several studies have indicated that CRS is associated with a failure of the mechanical and immunological barriers across the nasal mucosa^18,19^. Furthermore, patients with CRS have been shown to have impaired mucociliary function^20^, which increases susceptibility to respiratory infections^21^. Second, there is biological plausibility for a relationship between CRS and pneumonia. *Haemophilus influenzae*, *Moraxella catarrhalis*, and *Streptococcus pneumoniae* are the primary respiratory tract pathogens involved in both acute and chronic rhinosinusitis^22^. *Staphylococcus aureus*, *S. epidermidis*, and anaerobic Gram-negative bacteria predominate in CRS^23^. Moreover, these are the main causative pathogens of community-acquired and hospital-acquired pneumonia^24^. Third, the effectiveness of the pneumococcal vaccination may be reduced in patients with CRS. CRS is known to be one of the most common manifestations of humoral immunodeficiency^25^. Specific antibody deficiency (SAD) is a primary immune deficiency associated with a qualitative defect in antibody function. SAD is defined as a poor antibody response to polysaccharide antipneumococcal vaccine, such as Pneumovax®, in the presence of recurrent or chronic sinopulmonary infections^26^. A meta-analysis reported a high prevalence of humoral immune deficiency in CRS, with 8–34% of subjects diagnosed with SAD^27^. Last, an increase in cytokines induced by CRS-related inflammation may promote systemic inflammation, including in the lung. However, it is necessary to determine whether CRS directly causes pneumonia, or whether both diseases are simply different manifestations of common pathophysiology.

The association between CRS and pneumonia was seen only in the younger age, female sex, and CRSsNP subgroups. Since the risk of pneumonia increases with age and the presence of comorbidities^24,28^, the effects of other risk factors, such as CRS, may be emphasized in the younger age group. A study showing increased odds of having community-acquired pneumonia in CRS with severe sphenoid involvement attributed this association to the posterior location with the possibility of postnasal drainage into the lower respiratory tract^11^. One of the explanations for the linkage between the upper and lower airways includes postnasal drainage of inflammatory material into the lower airway. A previous study evaluating sex-specific differences in CRS patients showed that women reported more problems with postnasal drainage than men^29^. While CRSwNP is characterized by eosinophil-related inflammation, Asian CRSsNP patients show prominent neutrophil-related inflammation^30^. Although neutrophils are essential components of the immune response to pulmonary infection, they can also be harmful to host tissue. Excessive neutrophil activation results in severe tissue damage as a result of the exacerbated release of toxic agents, including proteinases, cationic polypeptides, cytokines, and reactive oxygen species^31^. However, further studies may be required to clarify the pathophysiology behind this finding.

The present study has some limitations. First, the incidence of pneumonia was relatively low and there may be potential for diagnostic misclassification. However, in Korea, the National Health Insurance system covers almost the entire population and is characterized by cost effectiveness and easy access to medical institutions. Given the distinct clinical characteristics of lower respiratory tract infection, misclassification of patients with signs and symptoms of pneumonia into the nonpneumonic diagnostic category is unlikely^32^. Second, there was a possibility of unadjusted covariates because there were no data about pneumococcal vaccination, details of medication use such as corticosteroid or antibiotics, immunosuppression status, atopy, and allergies. However, we adjusted for several confounding factors, including asthma, COPD, and other comorbidities that could affect the risk of pneumonia. We used CCI, which is widely used to classify various comorbid conditions and underlying diseases, including diabetes, renal failure, any malignancy, and AIDS/HIV^33^. Third, the incidence rates of community-acquired and hospital-acquired pneumonia cannot be investigated separately. Nevertheless, the strength of this study is that it is a population-based study that revealed the relationship between CRS and pneumonia.

In conclusion, CRS patients were more likely to have pneumonia. Physicians should be aware of the possible risk of pneumonia in patients with CRS.

## Materials and methods

### Study population

We used data from the Korean National Health Insurance Service-Health Screening Cohort for this study, and a comprehensive explanation for this cohort is provided elsewhere^34^. This study was performed in accordance with relevant guidelines/regulations and approved by the Institutional Review Board (IRB) of Hallym University (IRB No: 2019-10-023). The need for written informed consent was waived due to its minimal risk involved in the retrospective analysis.

### Participant selection

**Figure 1:**
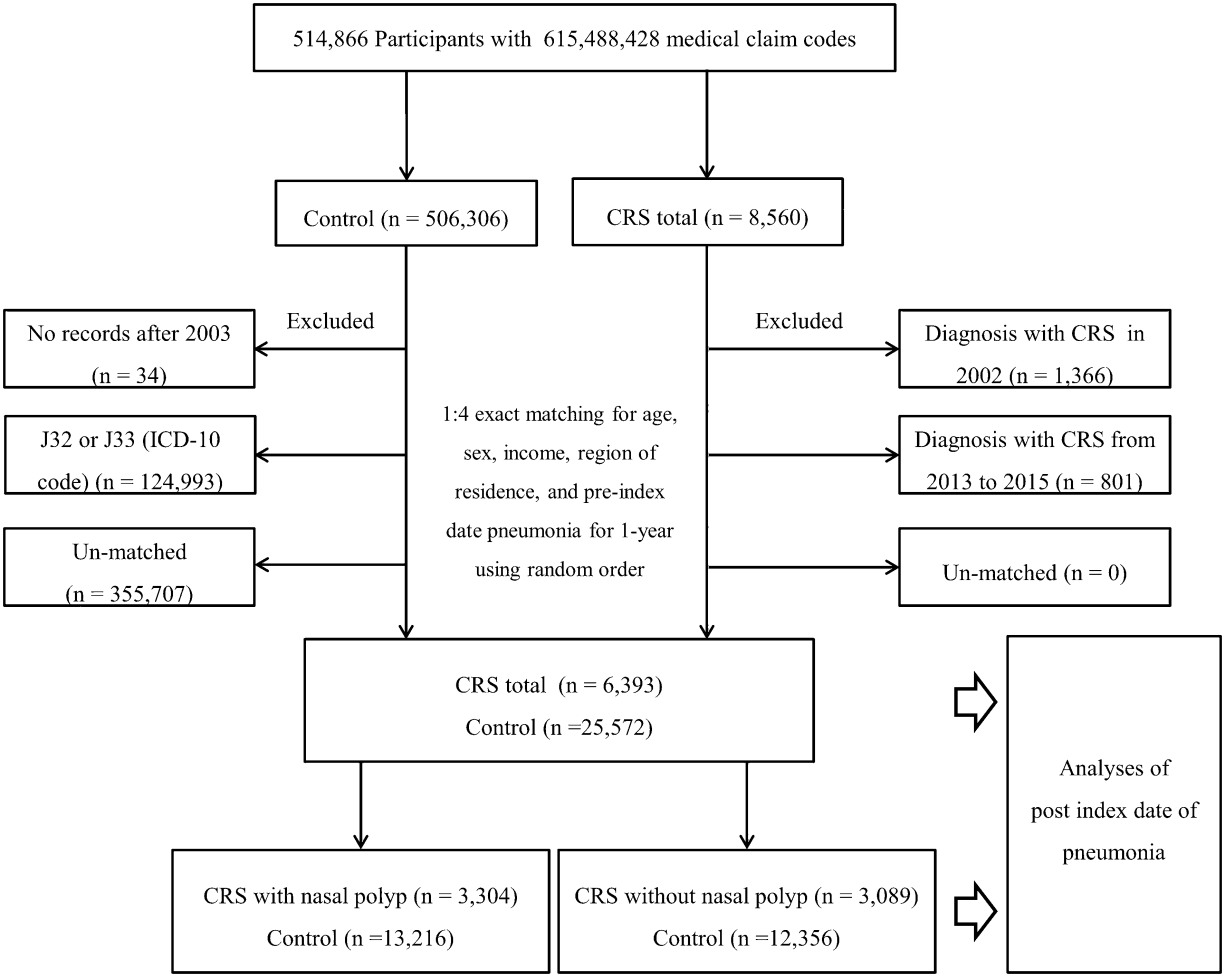
A schematic illustration of the participant selection process that was used in the present study. Of a total of 514,866 participants, 6,393 of the total CRS participants were matched with 25,572 control participants for age, sex, income, region of residence, and pneumonia for 1 year prior to the index date. Additionally, the final participants were further classified into two groups: CRS with nasal polyps (n = 3,304) with matched controls (n = 13,216) and CRS without nasal polyps (n = 3,089) with matched controls (n = 12,356).

Among the 514,866 participants with 615,488,428 medical claim codes, participants were selected as the CRS total group (n = 8,560) according to the definition in our previous study^35^. Participants without a history of CRS were selected as a control group (n = 506,306). Participants in the CRS total group who diagnosed with CRS in 2002 were removed (n = 1,366) to calculate the history of pneumonia from the date of first diagnosis of CRS (ID) to the date 1 year prior (pre-ID pneumonia for 1 year). In the control group, participants who died before 2003 or had no records after 2003 (n = 34) and those diagnosed with J32 (chronic rhinosinusitis) or J33 (nasal polyps) using the ICD-10 code at least once (n = 124,993) were excluded. If the participants were treated for the first time from 2013 to 2015, they were excluded from the CRS total group to count the diagnosis of pneumonia for 3 years following the ID (n = 801). The participants with CRS were 1:4 matched with the controls for age, sex, income, and region of residence. The CRS total and control groups were additionally matched by pneumonia incidence for 1 year pre-ID (0 times; ≥ 1 time). To minimize selection bias, the controls were selected with random number order. The ID of the controls was set as the ID of their matched participant with CRS. Therefore, each CRS participant and his or her matched controls had the same ID. During the matching process, 355,707 control participants were excluded. Finally, 6,393 of the total CRS participants were 1:4 matched with 25,572 control participants. Additionally, the final selected participants were classified into CRSwNP (n = 3,304) with matched controls (n = 13,216) and CRSsNP (n = 3,089) with matched controls (n = 12,356) (Fig. 1).

### Chronic rhinosinusitis (Exposure)

CRS with/without nasal polyps were defined using ICD-10 codes (J32, chronic sinusitis; J33, nasal polyp) and head and neck computed tomography evaluations following our previous study^35, 36^.

### Pneumonia (Outcome)

Pneumonia was identified based on ICD-10 codes (J12 to J18). Patients with these codes, who underwent chest X-ray (claim codes: G2101-G2105, G2111, G2112, G2121, G2201, G2301-G2305, G2322) or chest CT (claim codes: HA424, HA434, HA444, HA454, HA464, HA474) were defined as having pneumonia. Pneumonia for 1 year period pre-ID was categorized as 0 times and ≥ 1 time. The number of pneumonia diagnoses was counted from the ID to the date after the first year period (1-y post-ID pneumonia), second year period (2-y post-ID pneumonia), and third year period (3-y post-ID pneumonia).

### Covariates

Age groups were divided by 5-year intervals into 10 groups. Income groups were classified into 5 classes (class 1 [lowest income] – 5 [highest income]). The region of residence was grouped into urban and rural areas according to our previous study^37^. Tobacco smoking, alcohol consumption, and obesity were categorized in the same way as in our previous study^38^. Total cholesterol (mg/dL), SBP (mmHg), DBP (mmHg), and fasting blood glucose (mg/dL) were measured. The CCI^39^ score ranged from 0 (no comorbidities) to 29 (multiple comorbidities) except for COPD. Asthma and COPD were defined following our previous studies^40, 41^.

### Statistical analyses

The general characteristics of the CRS total and control groups were compared using standardized differences. Simple and multiple linear regressions were analyzed to calculate estimated values and 95% CIs for 1-y post-ID pneumonia, 2-y post-ID pneumonia, and 3-y post-ID pneumonia in the CRS total, CRSwNP, and CRSsNP groups compared to each matched control group. Both simple and multiple linear regressions were stratified by age, sex, income, and region of residence. In multiple linear regression, we used 2 models for analyses. Model 1 was adjusted for obesity, smoking status, alcohol consumption, total cholesterol, SBP, DBP, fasting blood glucose, CCI score, asthma history, and COPD history. Model 2 was adjusted for model 1 plus 1-y pre-ID pneumonia. For the subgroup analyses, we divided participants by age (< 60 years old and ≥ 60 years old) and sex (males and females). Simple and multiple linear regressions were calculated. All analyses were two tailed, and significance was defined as P-values less than 0.05. SAS version 9.4 (SAS Institute Inc., Cary, NC, USA) was used for statistical analyses.

## Data Availability

Data in this study were from the Korean National Health Insurance Service-Health Screening Cohort. Releasing of the data by the researcher is not allowed legally. All of data are available from the database of National Health Insurance Sharing Service (NHISS) (https://nhiss.nhis.or.kr/). NHISS allows all data for any researcher who promises to follow the research ethics with some cost. If you want to access the data of this article, you could download it from the website after promising to follow the research ethics.
